# Olfactory Receptors and Aortic Aneurysm: Review of Disease Pathways

**DOI:** 10.3390/jcm13247778

**Published:** 2024-12-19

**Authors:** Theodora M. Stougiannou, Konstantinos C. Christodoulou, Dimos Karangelis

**Affiliations:** Department of Cardiothoracic Surgery, University General Hospital, Democritus University of Thrace, 68100 Alexandroupolis, Greece; konstantinoschristodoulou@yahoo.gr (K.C.C.); dimoskaragel@yahoo.gr (D.K.)

**Keywords:** aortic, aneurysm, olfactory, odorant, cardiovascular, biology, pathophysiology

## Abstract

Aortic aneurysm, the pathological dilatation of the aorta at distinct locations, can be attributed to many different genetic and environmental factors. The resulting pathobiological disturbances generate a complex interplay of processes affecting cells and extracellular molecules of the tunica interna, media and externa. In short, aortic aneurysm can affect processes involving the extracellular matrix, lipid trafficking/atherosclerosis, vascular smooth muscle cells, inflammation, platelets and intraluminal thrombus formation, as well as various endothelial functions. Many of these processes are interconnected, potentiating one another. Newer discoveries, including the involvement of odorant olfactory receptors in these processes, have further shed light on disease initiation and pathology. Olfactory receptors are a varied group of G protein coupled-receptors responsible for the recognition of chemosensory information. Although they comprise many different subgroups, some of which are not well-characterized or identified in humans, odorant olfactory receptors, in particular, are most commonly associated with recognition of olfactory information. They can also be ectopically localized and thus carry out additional functions relevant to the tissue in which they are identified. It is thus the purpose of this narrative review to summarize and present pathobiological processes relevant to the initiation and propagation of aortic aneurysm, while also incorporating evidence associating these ectopically functioning odorant olfactory receptors with the overall pathology.

## 1. Introduction

Aortic aneurysm (AA) can be generally described as a localized dilation in the aorta, often differently defined depending on localization [1]; it can affect the thoracic and abdominal aorta separately or span both locations, and it can be associated with emergency and fatal sequelae (rupture) [2]. While AA age-adjusted mortality rates have been decreasing in Western Europe, they have been increasing in Central and Eastern Europe, at least up until 2019, as measured by relevant epidemiological studies [3]. The aorta is a layered structure composed of cells interacting with each other as well as the extracellular matrix (ECM) to maintain wall function and homeostasis. Differential embryologic origin of the cells and tissues composing the thoracic and abdominal aorta can often lead to variations in these homeostatic pathways and as such, variations in the pathobiological pathways observed upon perturbation [4]. Furthermore, genetic studies have implicated many genetic mutations, some of which differ between thoracic and abdominal aneurysms [5]. Despite these variations, however, it is generally understood that AA disease mechanisms in both thoracic and abdominal locations revolve around derangements in ECM homeostasis, lipid trafficking, vascular smooth muscle (VSMC) and endothelial cell function, inflammation, and thrombus formation as well as pathological angiogenesis [6].

Olfactory receptors in mammals are a diverse group composed of multiple receptor families. These range from odorant receptors in the olfactory mucosa and pheromone receptors in the vomeronasal organ of some animals, to receptors with functions not yet fully elucidated or characterized [7]. Most olfactory receptors in humans contribute to olfaction, though additional receptor families such as pheromone receptors have also been recognized [8,9,10,11,12]. Odorant olfactory receptors are mostly found on the surface of olfactory sensory neurons and aid in the recognition of volatile chemicals. Despite their classic localization and function associated with olfaction, many ectopically expressed odorant olfactory receptors have also been identified in a multitude of tissues, including the cardiovascular system [13]. These can contribute to many diverse functions, while disruption or perturbation in their expression can often lead to disease [14]. The contribution of odorant receptor activation or disruption in AA development will be thus examined, framing associated pathways within the context of existing AA pathobiology.

## 2. Olfactory Receptors

G protein-coupled receptors (GPCR) are the fourth largest protein superfamily in the human genome [15], with more than 800 encoded genes [16]. GPCR structure and signaling mechanisms are conserved across mammalian organisms, comprising seven transmembrane (7TM) α-helix domains with an extracellular amino terminus (N-terminal) and an intracellular carboxyl terminus (C-terminal) capable of binding heterotrimeric G proteins (G_αβγ_). GPCRs can be in turn classified into four classes (at least in humans), namely A, B, C [7] and F [17] and five subfamilies, each with a distinctly different structure. This includes families such as Glutamate, Rhodopsin, Adhesion, and Frizzled/Taste2 as well as Secretin receptors, with most GPCRs falling under the rhodopsin protein family category [18].

Mammalian olfactory systems comprise several sensory organs, including the main olfactory epithelium, the vomeronasal organ [19], the septal organ and the Grueneberg ganglion [20]. Olfaction thus depends on the recognition of varying chemicals such as odorants and pheromones, important, in turn, for the recognition of chemosensory information [7]. Associated receptors generally comprise various groups associated with chemosensation, including odorant olfactory receptors (class A GPCRs resembling rhodopsin receptors) and pheromone vomeronasal receptors (V1R, V2R), part of an accessory olfactory system (vomeronasal system) that is present in some animals (composed of class A and C GPCRs, respectively) [8,21]. Additional receptors involved in chemosensation include trace amine-associated receptors (TAAR) [22], formyl peptide receptors (FPRs) involved in inflammatory responses and allowing for the recognition of infected or contaminated substances [9], and finally, guanylyl-cyclase GC-D. Most of these receptors are GPCRs with seven TM domains, with only guanylyl-cyclase GC-D composed of only one TM domain and involved in guanylin, CO_2_ recognition [7]. While odorant olfactory receptors are quite extensive in humans, most *V1R* loci are pseudogenes, though some pheromone receptor genes have been identified in humans as well (mostly of the V1R, TAAR type) [10,23]. FPRs involved in human immune system processes have been identified as well [7,11,12].

In humans, odorant olfactory receptors (OR) are a member of the class A GPCR group, as previously detailed; they are usually referred to with the prefix ‘OR’, followed by a number denoting the family, a letter corresponding to the subfamily, and finally, another numeral representing the individual gene [14,24]. Human ORs, which have been found to be encoded by about 400 genes, are usually found on the surface of olfactory sensory neurons within the main olfactory epithelium, where they can come into contact with and aid in the identification of various odorant substances [17].

### 2.1. Olfactory Receptors: Structure and Function

Activation of an olfactory GPCR by a chemical odorant stimulates signaling pathways through the trimeric G_olf_ protein [25]. Each odorant can activate different ORs, though one OR can only be activated by a particular group of odorant molecules. Specific details about molecular structure have been elucidated through cryogenic electron-microscopy (EM) analysis of OR51E2 and its activation by propionate [26]. This receptor is also expressed ectopically, thus highlighting general aspects of ectopic OR structure and function as well. As with most similar receptors, ORs exhibit a structure composed of 7TM helices with an odorant-binding pocket between TM3-TM6 [17], protected from the main extracellular space [27]. Upon ligand binding, the receptor undergoes conformational changes, including outward rotation of TM6 towards the cytoplasm, possibly driven by sequence variations in extracellular loop 3 (ECL3); a cavity is thus created, allowing for the binding of G_olf_ [26].

Following binding of G_olf_, dissociation into α and βγ subunits occurs [17,28]. The G_αolf_ subunit then binds to and activates adenylate cyclase III in the cellular membrane, which is OR-specific, leading to ATP hydrolysis and production of cAMP. Intracellular cAMP then stimulates the opening of Na^+^/Ca^2+^ channels (CNG), which along with Ca^2+^-activated Cl^−^ channels, lead to membrane depolarization of the olfactory sensory neuron [17,29]. A signal is generated and traverses the olfactory neuron, generating an axon potential and allowing transmission towards the olfactory bulb and other relevant downstream anatomical structures [17,29,30].

### 2.2. Olfactory Receptors: Ectopic Receptors and the Cardiovascular System

Although ORs have been classically associated with olfaction, they have many other roles, perhaps owing to the large amount of variation found within their respective genetic loci [31]. Based on analysis of approximately 119,069 reported variants [32,33], *OR* loci are generally composed of many different variants and pseudogenes. These can be derived through different mechanisms, including missense mutations, which are the most common, frameshift mutations, and changes in the 3′ UTR and 5′ UTR regions [31]. The first study detailing ectopic OR expression in mammalian germ cells was published in 1992 [34]; ever since, ectopic expression of *OR* genes has been well-reported, with studies revealing many different localizations, including the hematopoietic and cardiovascular systems [14]. Based on NGS-based transcriptome analysis, there are 11 *OR* genes expressed in the human heart, including *OR51E2*, *OR51E1*, *OR52N4*, *OR13A1*, *OR2H2*, *OR10AD1*, *OR3A3*, *OR52B6*, *OR2K2*, *OR8G5*, *OR4D6*, and *OR10J5* [35], although many more pseudogenes have been reported as well [36].

The receptor OR51E1 (Olfr558) has been found to be the most highly expressed OR in both adult and fetal cardiomyocytes; its function is mainly associated with the recognition of medium-chain fatty acids (MCFA) (i.e., nonanoic, dodecanoic, tetradecanoic acid) [37,38]. Ligand binding and receptor activation induce a negative chronotropic effect via the reduction of Ca^2+^ transients and thus, intracellular Ca^2+^. There is also an associated reduction in cardiomyocyte contractility (negative inotropic effect). All these effects are mediated through cAMP-induced stimulation of Na^+^/Ca^2+^ channels (cyclically activated nucleotide-gated channels-CNG). Epicardial adipocytes can further affect cardiac contractility via the storage and secretion of additional MCFAs [39,40], which function as OR51E1 ligands. While the favorable effects of MCFAs on cardiac metabolism, post-injurious contractile function and diabetic cardiomyopathy are well known [40,41], the specific function of MCFA-mediated OR51E1 activation in this context has not been fully elucidated [37].

The receptor OR10J5 (Olfr16) has been isolated from samples procured from the human aorta, coronary arteries, and human umbilical vein endothelial cells (HUVEC), suggesting a possible role for OR10J5 in the regulation of endothelial cell behavior [35]. In HUVECs, OR10J5 activation induces migration via Ca^2+^-dependent Protein kinase B (PKB or AKT) signaling. When experimentally stimulated with lyral, an OR10J5 agonist, angiogenesis occurs in murine models [35,42]. The basis of OR10J5 receptor activation revolves around modulation of endothelial cell cytoskeleton, brought on by an increase in intracellular Ca^2+^, in turn leading to phosphorylation of downstream signaling proteins such as AKT and Extracellular signal-regulated kinase (ERK). Eventually, migration of endothelial cells occurs, contributing to angiogenesis [43]. Angiogenesis is mediated via the downregulation of endothelial cell–cell junctions due to various effects on the actin cytoskeleton, including an increase in cortical rim actin (actin polymerization) and changes in microtubule arrangement (microtubule depolymerization) associated with adherens junctions [44,45]. Eventually, changes in endothelial cell morphology occur, modulating not only barrier activity [46] but possibly contributing to processes such as angiogenesis as well [35].

The receptor OR51E2 (Olfr78) has been found to be expressed in VSMCs of small resistance vessels in various locations, including the heart and skin; it is activated by propionate, a substance often produced by gut microbiota, and contributes to arterial pressure modulation in these vessels. More specifically, propionate-induced OR51E2 activation acts as a counterregulatory mechanism for the hypotensive effects of short chain fatty acids (SCFA), such as propionate, on other receptors, modulating tissue perfusion [47,48]. In murine studies, the *OR51E2* ortholog, *Olfr78*, has been identified in small resistance vessels in the kidney and in particular, the juxtaglomerular afferent arteriole involved in renin secretion. The receptor Olfr78 in the juxtaglomerular apparatus (JGA) is activated by SCFAs such as acetate and propionate, substances normally produced by gut flora. In response to this activation, there is an increase in intracellular cAMP causing the release of renin and contributing to blood pressure regulation, achieved mainly via counterregulation of the hypotensive effect mediated via propionate interaction at other receptors [49]. This is further corroborated by studies in which homozygotic *Olfr78* knockout (KO) increases susceptibility to the hypotensive effects of propionate; in these studies, blood pressure decrease is mediated via propionate-induced activation of Gpr41 and Gpr43. The latter are normally found in arteries of the kidney and other locations (aorta, iliac artery) [50,51,52].

**Figure 1:**
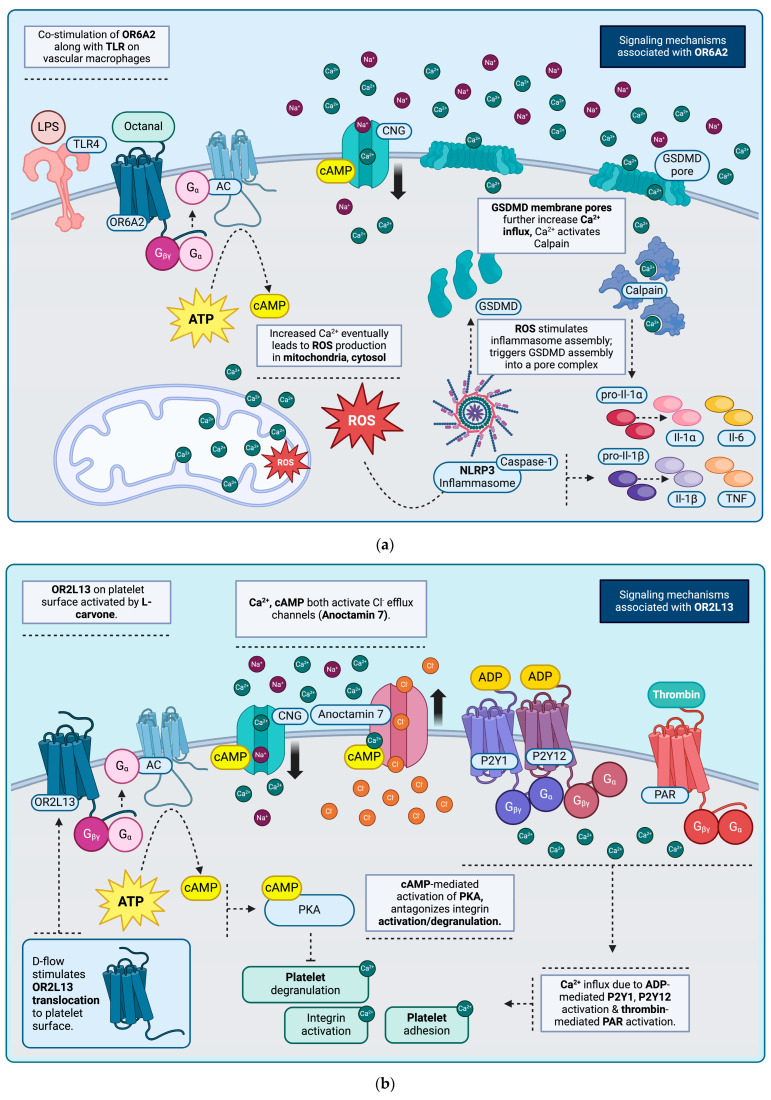
Olfactory receptor signaling mechanisms associated with aortic pathophysiology. (**a**) Signaling mechanisms associated with OR6A2 activation. (**b**) Signaling mechanisms associated with OR2L13 activation (created in BioRender.com, accessed on 18 November 2024) [53,54,59]. OR6A2, Olfactory receptor family 6 subfamily A gene 2; LPS, Lipopolysaccharide; TLR4, Toll-like receptor 4; AC, Adenylate cyclase; ATP, Adenosine triphosphate; CNG, Cyclically activated nucleotide gated channels; Na^+^, Sodium; Ca^2+^, Calcium; ROS, Reactive oxygen species; NLRP3 Inflammasome, NLR family pyrin domain containing 3 inflammasome; GSDMD, Gasdermin D; pro-IL-1β, pro-Interleukin one-beta; IL-1β, Interleukin one beta; pro-IL-1 α, pro-Interleukin one-alpha; IL-1α, Interleukin one-alpha; IL-6, Interleukin-6; TNF, Tumor necrosis factor; oxLDL, oxidized Low-density lipoprotein; OR2L13, Olfactory receptor family 2 subfamily L gene 13; cAMP, cyclin Adenosine monophosphate; Cl, Chloride; Na, Sodium; PKA, Protein kinase A; PAR1, Protease-activated receptor type 1. (**a**) Created in BioRender. Stougiannou, T. (2024) https://BioRender.com/e60l756 (accessed on 1 November 2024); (**b**) Created in BioRender. Stougiannou, T. (2024) https://BioRender.com/t03c015 (accessed on 1 November 2024).

Olfr2 receptors have been isolated from Cluster of differentiation 45 (CD45)+ F4/80+ vascular macrophages derived from murine aortic specimens as well as CD45- Cluster of differentiation 31 (CD31)+ endothelial cells (ECs) and CD45- CD31-VSMCs. In addition, the human ortholog *OR6A2* has also been found to be expressed in human aortic specimens and associated vascular macrophages as well [53]. In both human and murine species, the receptors Olfr2 and OR6A2 can be activated by octanal, a constituent of oxidized Low-density lipoprotein (oxLDL), derived from octanoic and oleic acid via lipid peroxidation. Both lipid peroxidation as well as oxLDL are characteristic of atherosclerotic aortic lesions [53,54,55,56]. Receptor activation generally comprises a complex interplay between atherosclerosis mediators and plaque constituents (octanal) as well as inflammatory mediators (lipopolysaccharide (LPS)), eventually causing aortic wall inflammation [56]. It seems that both LPS along with octanal are required for OR6A2 receptor activation, which will then lead to cAMP-mediated activation of CNG channels and, as a result, an increase in intracellular Ca^2+^. In vascular macrophages derived from atherosclerotic aortas, however, appropriate receptor activation occurs without LPS supplementation, as other endogenous atherogenic TLR ligands [54] along with octanal can produce the same effect. OR6A2 activation eventually leads to upregulation of mitochondrial and cytosolic reactive oxygen species (ROS), inducing the assembly of the NLR family pyrin domain containing 3 (NLRP3) inflammasome. Activation of caspases, as a result, leads to the cleavage and thus activation of Interleukin-1 beta (IL-1β) as well as indirect activation of Interleukin-1 alpha (IL-1α) via calpain and Gasdermin D (GSDMD) [57]. The latter involves GSDMD-mediated plasma membrane pore formation, allowing for Ca^2+^ influx and calpain activation. Calpain will then activate IL-1α via proteolysis [58]. Additional inflammatory cytokines induced as a result of this action include Tumor necrosis factor (TNF) and Interleukin-6 (IL-6) [53]. Finally, the OR itself has been shown to be upregulated after administration of LPS with octanal, possibly due to the LPS-induced TLR4 receptor activation and subsequent nuclear translocation of Nuclear factor kappa-light-chain-enhancer of activated B cells (NF-κB) and Activating protein-1 (AP-1) [53,54] (Figure 1a).

The gene products of *OR2L13* (and its murine ortholog *Olfr168*) have been identified on platelet and megakaryocyte surfaces. Megakaryocytes, in particular, can carry additional *OR* gene transcripts, though during the course of their lifespan, most of these are eventually lost, with only three ORs eventually identified on the surface of circulating platelets. One of these is OR2L13 [59], stored within platelet α-granules and co-localizing with P-selectin. Though OR2L13 expression remains the same despite hemodynamic variations, under turbulent (disturbed) flow conditions, it converges in the central platelet granulomere and translocates to the surface [59,60]. OR2L13 can be upregulated by several different agonists, including carvone, a terpene compound and ingredient in spearmint, found in two enantiomer conformations [61]. Both (+) and (−) enantiomers can activate the receptor, although the (−) enantiomer produces a stronger effect. Once active, adenylate cyclase (AC) is recruited, cleaving ATP into cAMP and allowing it to activate a membrane CNG channel along with various other targets, including Protein kinase A (PKA). CNG stimulation then leads to Ca^2+^ influx. In the pathway initiated by OR, Ca^2+^ along with cAMP will eventually activate another membrane channel, Anoctamin 7, stimulating Cl^−^ efflux as well as PKA. This inhibits platelet degranulation and eventually prevents platelet aggregation as well [59]. OR activation in platelets under turbulent flow can thus modulate other mechanisms that cause platelet aggregation, due to the Ca^2+^-mediated stimulation of platelet degranulation and adhesion [59,62] (Figure 1b) (Table 1).

## 3. Aortic Aneurysm

### 3.1. Aortic Aneurysm: Definitions

The aorta is the largest blood vessel [66], with an average diameter of about 3.0 cm, though size may vary with sex, age and body type [67]. It is composed of three layers: an inner intima with an endothelial cell lining, a media composed mainly of elastin/collagen fibers and VSMC, and finally, an outer adventitia composed of connective tissue. As expected of an elastic-type artery, it has a much higher elastin content in the tunica media, as opposed to other arteries [68]. Often, due to a combination of genetic and environmental factors, dilatations along its length can occur, which will eventually cause aneurysm formation after reaching a particular size. Depending on localization, definitions vary; in the ascending thoracic aorta, aneurysm is defined as vessel diameter greater than 4.5 cm, while in the descending thoracic and abdominal aorta, aneurysm is usually defined as vessel diameter greater than 1.5 times the initial diameter [2]. AA is one of the most common diseases affecting the aorta, second only to atherosclerosis [1,69].

Disease classification is based on anatomic location, with thoracic aortic aneurysms (TAAs) observed in the thoracic aorta (affecting anywhere from the aortic root, arch and descending thoracic aorta), abdominal aortic aneurysms (AAAs) identified in the abdominal aorta, and thoracoabdominal aneurysms (TAAAs) spanning both sections [1,2]. In addition, AAAs may also be classified according to location, though most seem to form in the infrarenal aorta [70].

### 3.2. Aortic Aneurysm: Etiology

#### 3.2.1. Genetic Factors in Thoracic and Abdominal Aortic Aneurysm

TAAs can generally be attributed to genetic, congenital, and environmental factors; in fact, up to 25% of all TAAs are due to genetic causes, some of which comprise various genetic syndromes (syndromic TAA). Furthermore, while only 20% of the genetic disease states associated with TAA display autosomal dominant inheritance, none of the genes exhibiting a causative relationship with TAA are associated with AAA [5]. Syndromes associated with TAA include Marfan Syndrome (MFS) [71], Ehlers–Danlos Syndrome (EDS) [72], Cutis Laxa Syndrome (CLS) [73], Congenital Contractural Arachnodactyly (CCA) [74], Loeys–Dietz Syndrome (LDS) [75], Juvenile Polyposis (JPS)/Hereditary Hemorrhagic Telangiectasia (HHT) [76], multisystemic smooth muscle dysfunction syndrome [77] and Cardiac valvular dysplasia (CVD) [78,79,80]. Non-syndromic TAA is usually due to standalone genetic mutations, some of which are due to familial inheritance or de novo appearance [81].

For many of the above syndromes as well as sporadic and non-syndromic thoracic aortic disease, involved genes usually relate to processes associated with aortic wall homeostasis. As such, affected genes include those associated with VSMC function/survival, such as *smooth muscle actin α2* (*Acta1*), *smooth muscle myosin heavy chain 11 (Myh11)*, *myosin light chain kinase (Mylk)*, *filamin A (Flna)* and Transforming growth factor beta (TGFβ) signaling pathways, including mutations in *TGFβ receptor type I (Tgfbr1)/type II (Tgfbr2)*, *TGFβ2 (Tgfb2)* and *Mothers against decapentaplegic drosophila homolog 3 (Smad3). Tgfbr1/Tgfbr2*, *Tgfb2* and *Smad3* are usually affected in LDS, while *Smad4* is affected in JPS/HHT [82]. Additional genes include those involved in ECM function and homeostasis, including *Fibrillin-1 (Fbn1)*, *Lysyl oxidase (Lox)*, *Biglycan (Bgn)* associated with MFS [83], *Procollagen type III α1 (Col3a1)* associated with vascular EDS and *Elastin (Eln)* usually associated with CLS [84,85].

Genes relevant to S-adenosylmethionine (SAM) synthesis, such as *Methionine adenosyltransferase II α* (*Mat2a*), have also been identified, usually involved in metabolic reactions with nitrogenous compounds (protein, deoxyribonucleic acid (DNA), ribonucleic acid (RNA)). Though relevant mutations have been uncovered in genetic studies [5], with animal studies showcasing a role for SAM in the mitigation of aortic dissection via inhibition of VSMC phenotypic switch [86] and inflammation [87], more research may be needed to uncover additional roles or frame these functions within the general context of AA pathobiology. Additional studies have further unveiled associations between *Ariadne drosophila homolog 1* (*Arih1*) and Ascending aortic disease/Aortic dissection [88,89]. Arih1 is a protein associated with the Linker of nucleoskeleton/cytoskeleton protein complex (LINC), which binds structural proteins (lamins, nestrins) and cell surface integrins, allowing for modulation of gene transcription, chromatin modification and cellular phenotypic switch [5,90,91]. In the case of AA, LINC functions by linking the nucleus with the VSMC contractile unit and extracellular fibrillin-1. Any derangements in any of these cellular structures eventually contribute to disruption of tissue architecture and thus, disease [89].

AAAs also have a genetic component, with about 20% of patients reporting a first-degree relative. Genetic etiologies for AAA usually follow an autosomal dominant pattern of inheritance, though there seems to be evidence for other modes of inheritance as well, including combined dominant/recessive inheritance, along with evidence of clustering within families [5,92]. Syndromic AAA, as opposed to TAA, is comparatively rarer, though cases of MFS-associated AAA have been reported [93,94,95]. In addition, genetic analyses along with genome-wide association studies (GWAS) have revealed several genetic loci comprising genes and non-coding areas associated with AAA, many of which affect MMP enzymes, ECM components or inflammatory pathways [96]. Examples include *Cdkn2b-As1/Anril*, an antisense RNA [97,98] that modulates the expression of Cyclin-dependent kinase inhibitor 2b (Cdkn2b) [5,96] and DAB2 interacting protein (Dab2ip) [99]. Both are associated with inflammation and vascular proliferation [5,100]. Additional genetic loci associated with aortic disease include *Interleukin-6 receptor (Il6r)* [101] and *Low-density lipoprotein receptor-related protein 1 (Lrp1)* [102] associated with contractile function of VSMCs [103] and angiotensin signaling modulation [104]. Angiotensin modulation in turn regulates ECM homeostasis owing to the regulation of VSMC matrix metalloproteinase 9 (MMP9) expression [105].

Additional relevant variants that when mutated can lead to aortic disease include *MYND* (*myeloid-nervy-DEAF1*) *domain containing 2* (*Smyd2*) associated with VSMC myofibrillar structure [106] and macrophage-associated cytokine production [107]. The *Transcriptional regulator ERG* (*Erg*) gene often found in endothelial cells, loss of which may lead to endothelial-to-mesenchymal transition (EndMT) [108], is associated with angiogenesis, inflammation and embryonic development of the aorta. *Erg* derangements thus also contribute to aneurysm [5,109,110,111]. There are also many affected loci associated with genes involved in lipoprotein handling and metabolism in AAA, including the *Sortilin-1 (Sort1)* gene associated with plasma low-density lipoprotein (LDL)/cholesterol handling [112] and the *LDL/cholesterol receptor (Ldlr)* gene associated with LDL/Cholesterol handling [5,111,113] (Table 2).

#### 3.2.2. Non-Genetic Factors in Thoracic and Abdominal Aortic Aneurysm

Additional etiological factors for TAA include diseases/processes that increase aortic wall stress such as hypertension [114], smoking [115], cocaine abuse [116] and various forms of congenital disease such as coarctation of the aorta [117] and bicuspid aortic valve (BAV) [118]. As opposed to AAA, in the thoracic aorta, unless no other risk factors/acute events are identified, distribution between men and women is approximately equal [119]. Processes that affect tunica media are also be to blame, including age-associated degeneration [120], various inflammatory conditions such as atherosclerosis [121], infectious aortic disease (syphilitic, mycobacterial, bacterial and fungal aortitis) [122] and non-infectious aortitis such as Giant-cell arteritis (GCA) [123], Takayasu arteritis (TA) [124], sarcoidosis [125], Granulomatosis with polyangiitis (GPA) [126], Rheumatoid arthritis (RA) [127], Ankylosing spondylitis (AS) [128], Systemic lupus erythematosus (SLE) [129] and many others [81].

As with TAA, non-genetic risk factors can contribute to AAA as well, including atherosclerosis which has been found to increase risk for TAA as well [121]. Inflammatory processes and autoimmunity have also been implicated, including TA [124], GCA [130], AS [128], IgG4-related vasculitis [131], Inflammatory Abdominal Aortic Aneurysm (IAAA) within the spectrum of chronic idiopathic periaortitis/idiopathic retroperitoneal fibrosis [132] as well as infectious aortitis [133,134]. Though many types of non-infectious aortitis affect both the thoracic and abdominal aorta, there is some difference in the tendency of each disease to primarily affect primarily one location over the other. As such, while GCA tends to manifest more often in the thoracic rather than the abdominal aorta, the opposite holds true for IgG4-related vasculitis [131].

Smoking can also be implicated as an etiological factor, as nicotine and nitrites associated with tobacco smoke are detrimental to normal aortic wall structure [135]. Nicotine upregulates matrix metalloproteinase (MMP) activity, while tyrosine nitration can render native proteins antigenic, possibly uncovering a role for autoimmunity as well [136,137,138]. Additional risk factors include age and the male sex [119], hypertension, and coronary artery disease (CAD) [139]. Diabetes mellitus (DM), on the other hand, normally a risk factor for many cardiovascular entities, has been negatively associated with AAA [140]. Many antibiotics, including fluoroquinolones, have been implicated as well, particularly in abdominal or iliac artery aneurysms [141], most likely due to a mechanism involving MMP upregulation [142] (Table 3).

## 4. Aortic Aneurysm Pathophysiology

Pathobiological processes leading to AA development are quite complex, with new discoveries constantly being generated, including studies associating ectopic OR activation with AA pathways [53,59,65]. Pathways that contribute to AA emergence/progression can involve cells and factors in all three layers of the aorta, generating a complex interplay of pathophysiological processes, eventually leading to the establishment and further progression of disease [6].

### 4.1. Extracellular Matrix (ECM)

One group of pathobiological processes involved in AA development is related to ECM derangements and namely the interplay between extracellular collagen and elastin fibers. ECM composition and aortic wall thickness vary from region to region; there is a higher proportion of elastin in the thoracic aorta, allowing for the dampening of the left ventricular (LV) pressure wave, while collagen concentrations remain relatively constant, resulting in decreasing elastin-to-collagen ratios from thoracic to abdominal aorta [70]. Furthermore, ECM in the aortic wall exists within a state of constant remodeling, owing to the action of various metalloproteinases, including MMP, A disintegrin and metalloproteinase enzymes (ADAMs), ADAMs with thrombospondin motifs (ADAM-TS) and serine/cysteine proteases, including cathepsins and granzymes. In turn, activity of these enzymes is regulated via various protein inhibitors, including tissue inhibitors of metalloproteinases (TIMP) and α2-macroglobulins [70,144].

Under aneurysmal conditions, the above processes become deranged, leading to excessive ECM degradation due to increased MMP production. This usually occurs in response to inflammatory stimuli, with inflammatory cells themselves (macrophages, neutrophils) also producing MMPs and further contributing to ECM degradation. While pathological MMP activity is associated with both TAA and AAA, ADAM-TSs have been most extensively studied in TAA models, ADAMs in both TAA and AAAs and cathepsins mostly in AAAs [70]. Enzyme activity can be regulated through TIMP proteins; reduced expression as well as deficiency are usually to blame for progression of the disease, though some TIMPS such as TIMP3 are often overexpressed, creating a counterregulatory mechanism that exerts protective effects safeguarding against aortic dilatation [70,145,146].

ECM composition can also be altered, with elastin fragmentation and various structural derangements identified as the disease progresses. This is often due to *Fbn1* mutations leading to TGFβ signaling disruption, as fibrillin proteins normally modulate TGFβ signaling [147,148]. However, TGFβ signaling disruption may also be the initiating event, eventually leading to derangements in ECM composition. Inappropriate collagen levels [149], disruptions in the collagen assembly process, particularly during assembly of the collagen triple helix [150] and cross-linking, due to lysyl oxidase (LOX) downregulation or inactivity, have also been identified [151]. There are also disruptions in proteoglycan concentrations in the ECM; often, there is increased production of versicans and aggrecans, commonly observed in TAA [152]. In AAA models on the other hand, glycoproteins such as versican, perlecan and aggrecan are reduced, in turn affecting attachment, migration and proliferation of VSMCs [153]. Finally, there is accumulation of fibronectin and thrombospondin; thrombospondin normally facilitates mechanotransduction and organization of collagen and elastin fibers, along with fibronectin [154]. In addition, thrombospondin modulates angiogenesis by functioning as an anti-angiogenesis factor [155]; thrombospondin accumulation can thus impede processes such as angiogenesis, normal cell adhesion and proliferation [70,156,157,158] (Table 4).

### 4.2. Lipid Trafficking and Atherosclerosis

Mutations in genes relevant to lipid trafficking and metabolism have also been identified in relevant genetic studies, representing an important aspect in AA pathogenesis [5,159]. Mutations in the *Low-density lipoprotein receptor* (*Ldlr*) gene are generally seen in various dyslipidemic states such as familial hypercholesterolemia [160], type III hyperlipidemia [161] and most recently, within a subset of mutations that can increase susceptibility to aneurysm development, found at 19p13.2 genetic locus [5,113].

Low-density lipoprotein (LDL) is a circulating protein that facilitates the distribution of lipid particles to various locations; it comprises apolipoprotein B (ApoB) and cholesterol (CH) and can contribute to CH deposition within the arterial wall [162]. LDLR normally aids in lipoprotein particle clearance from the circulation, via apolipoprotein E (ApoE). *ApoE* gene variants thus modulate lipoprotein particle clearance rate and as such, LDL/CH levels, with some alleles conferring a higher risk for dyslipidemia and states of vascular inflammation such as coronary artery disease (CAD) [163]. Other apolipoprotein variants, on the other hand, assume a more protective role [164]. More specifically, *ApoE* variants can modulate risk for aortic aneurysm disease, with *E3/E4* genotypes mitigating aneurysmal expansion and homozygous *E3/E3* and *E2/E2* genotypes increasing associated risk [165]. Mendelian randomization and genome-wide association studies (GWAS) have further shown an association between LDLR, lipid trafficking and AAA development [166,167]. The very fact that LDLR KO organisms are often used for AAA modeling further indicates the contribution of disruption of LDL/CH trafficking to the overall process [5,113].

*Lrp1* gene mutations have also been implicated in TAA and AAA development; *Lrp1* generally encodes for the LDL receptor-associated protein 1 (LRP1), an endocytic transmembrane receptor protein found in many different cell types such as VSMCs, macrophages and fibroblasts. Though similar to other LDL receptors, it is larger and possesses functions unrelated to lipid metabolism, as it has been associated with VSMC contractility [103], regulation of MMP9 activity [102,168] and modulation of angiotensin signaling [102,104]. Studies have further shown LRP1 to be an important contributor to aortic wall integrity, particularly in the presence of high circulating lipid levels, a function mediated via Platelet-derived growth factor receptor beta (PDGFRβ) signaling [169]. This eventually affects actin organization and VSMC migration and as a result, structure and function of the aortic wall [5,170]. Finally, Lipoprotein A (LPA), normally associated with lipid trafficking and metabolism, has also been shown to exhibit a strong causal association with AA. It is thus clear that there is an association between genetically inherited dyslipidemia states and AAA [159].

#### Olfactory Receptors, Lipid Trafficking and Atherosclerosis

Activation of vascular macrophage surface ORs is another recently identified cause of vascular wall inflammation, which can further increase risk for AA progression. This occurs not only due to the ensuing inflammation itself, but also due to the association with lipid oxidation metabolic products (oxLDL) and lipid trafficking dysregulation, two pathways that both contribute to AA development. As previously stated, CD45+ F4/80+ vascular macrophages in the aorta can express odorant olfactory receptors, namely OR6A2 (Olfr2 in murine macrophages) [53]. OR6A2 (Olfr2) receptor activation in these cells triggers ROS production, inflammasome assembly and the production of inflammatory cytokines (IL-1β, IL-1α) [171]. Receptor activation in vascular macrophages has been achieved via octanal, a substance often derived from oleic acid peroxidation; in humans, octanal can be derived from oxLDL and correlates with total CH and High-density lipoprotein (HDL)/CH levels [56,172]. Octanal also has been identified as a component of murine atherosclerotic aortas [54]. Experiments in *ApoE* deficient (*Apoe*−/−) mice supplemented with octanal not only result in increased TNF and IL-1β production, as would be expected from Olfr2 activation, but atherosclerotic plaque size as well. While this event is reversible with receptor antagonism, it does not reduce systemic lipid levels themselves. These observations further corroborate the correlation between octanal and lipoprotein trafficking and their effect in atherosclerotic lesions, all mediated by OR6A2 (Olfr2) receptor activation [56].

**Figure 2:**
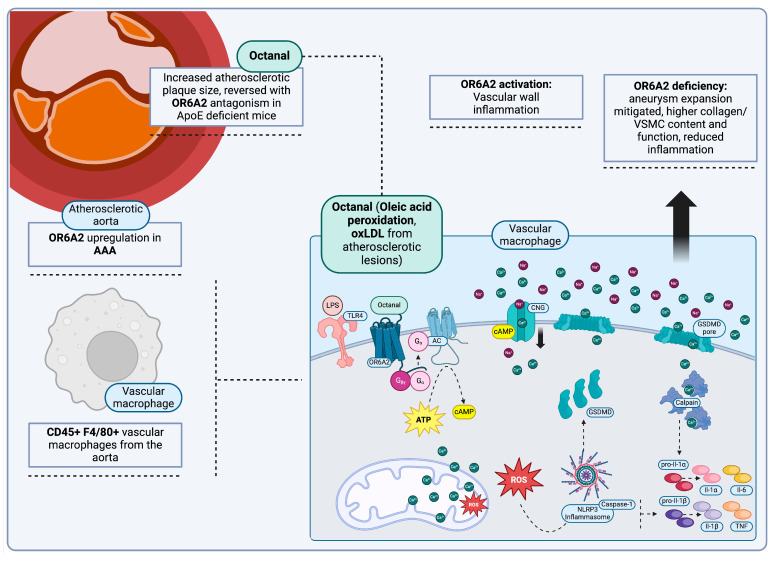
Signaling mechanisms associated with OR6A2 activation in the scope of aortic atherosclerosis and aneurysm formation. Octanal derived from oxLDL activates OR6A2 receptors on the surface of CD45+ F4/80+ vascular macrophages in the aorta. This in turn leads to production of inflammatory mediators via a process involving increased intracellular cAMP and NLRP3 inflammasome activation, contributing to vascular wall inflammation. Octanal administration in ApoE deficient murine models contributes to expansion of atherosclerotic plaque lesions, while OR6A2 antagonism reverses plaque formation but does not affect systemic lipid levels. Additional experiments examining OR6A2 deficiency have shown mitigation of the observed inflammation, increased VSMC and collagen content with improved VSMC function. Furthermore, increased OR6A2 levels have been found in specimens of AAA, while OR6A2 mitigates aneurysm progression (created in BioRender.com, accessed on 1 November 2024) [53,54,64,65,173]. OR6A2, Olfactory receptor family 6 subfamily A gene 2; LPS, Lipopolysaccharide; TLR4, Toll-like receptor 4; AC, Adenylate cyclase; ATP, Adenosine triphosphate; cAMP, cyclin Adenosine monophosphate; CNG, Cyclically activated nucleotide gated channels; Na^+^, Sodium; Ca^2+^, Calcium; ROS, Reactive oxygen species; NLRP3 Inflammasome, NLR family pyrin domain containing 3 inflammasome; GSDMD, Gasdermin D; pro-IL-1β, pro-Interleukin one-beta; IL-1β, Interleukin one beta; pro-IL-1 α, pro-Interleukin one-alpha; IL-1α, Interleukin one-alpha; IL-6, Interleukin-6; TNF, Tumor necrosis factor; oxLDL, oxidized Low-density lipoprotein; ApoE, Apolipoprotein E; CD45, Cluster of differentiation 45; Na, Sodium; VSMC, Vascular smooth muscle cell. Figure 2. Created in BioRender. Stougiannou, T. (2025) https://BioRender.com/l48w107 (accessed on 1 November 2024).

This association between OR6A2 and aortic aneurysm is corroborated further by studies showing an upregulation of these receptors in aneurysmal tissues, including human AAA specimens. In particular, deficiency of the *OR6A2* ortholog, *Olfr2*, in murine models mitigates aneurysm development and progression. Not only is aneurysm progression mitigated in Olfr2-deficient mice, but collagen and VSMC content are higher as well, with associated preservation of VSMC contractile activity, thus alleviating aspects of detrimental aortic remodeling. Inflammatory cell infiltrate is also reduced. It is thus evident that Olfr2 deficiency overall mitigates aortic remodeling, inflammation and aneurysm progression [64,65,173] (Figure 2).

### 4.3. Vascular Smooth Muscle Cells (VSMCs)

Another group of processes contributing to aneurysm formation centers around the survival and function of vascular smooth muscle cells (VSMCs), normally found in the tunica media. Under physiological conditions, VSMCs are usually quiescent with a mainly contractile phenotype; in response to various stimuli, they can undergo phenotypic switch characterized by secretory, proliferative as well as migratory features [174]. Some studies show that TGFβ signaling can aid in the maintenance of this contractile phenotype, as stimulation of VSMCs with TGFβ has been shown to upregulate expression of various genes associated with the contractile phenotype [175]. Other studies, however, make the case for TGFβ-based signaling contributing to aneurysm development as well [176,177,178]. The switch between different VSMC phenotypes is usually brought on by upregulation of Krüppel-like factor 4 (Klf4) along with downregulation of Myocardin (Myocd) and Serum response factor (SRF) via epigenetic remodeling. In turn, genes associated with the contractile phenotype are suppressed, while phagocytic-like markers are upregulated, owing to the concurrent release of de-differentiation stimuli such as platelet-derived growth factor-BB (PDGF-BB) and oxidized phospholipid molecules [179,180].

Many different VSMC subtypes have been identified, based on data from lineage tracing, single-cell transcriptomics and RNA sequencing; contractile phenotypes are identified in most specimens, while others, such as inflammatory VSMCs, appear primarily in murine specimens [181]. In human aortic specimens, five main VSMC phenotypic groups have been identified, including contractile VSMCs, stressed VSMC phenotypes, VSMCs with primarily proliferative characteristics upregulating both synthetic and contractile genes, a fibromyocyte VSMC group expressing mainly ECM genes [182,183] and a degradative VSMC phenotype exhibiting both proteolytic and phagocytic characteristics upregulated by mammalian Target of rapamycin (mTOR) [184]. Additional VSMC states include a mesenchymal stem cell (MSC)-like phenotype, mainly observed in patients with atherosclerosis and aortic aneurysm. In such cases, the phenotype switch occurs via the combined effects of TGFβ signaling downregulation and CH on Klf4, resulting in upregulation of the latter [185]. In addition, VSMCs in TAA samples from patients with MFS have been found to express stem cell markers [186], while there is also evidence for osteoblastic VSMC phenotypes contributing to medial calcification. The transition, in this case, is also regulated by Klf4, with osteoblastic VSMCs secreting alkaline phosphatase (AlkP) and osteopontin, leading to mild/moderate medial degeneration, elastin fragmentation and increasing risk for aortic wall rupture. Conversely, severe medial degeneration is associated with reduced rates of tunica media calcification [187,188].

All these VSMC subgroups point to a general plasticity of the VSMC phenotype, which can eventually lead to dysregulation of the normal aortic wall homeostasis. It is worth noting, however, that the specific contribution of each of these phenotypes in aortic aneurysm has not been fully elucidated [6]. Many different factors can contribute to phenotypic switch, one of which is aldehyde dehydrogenase 2 (ALDH2), an enzyme often associated with aortic endothelial dysfunction [189]. Experimental ALDH2 deficiency has been shown to allow the myocardin-dependent upregulation of contractile gene transcription via inhibition of miR-31-5p processing. As a result, retainment of the contractile phenotype and prevention of phenotypic switch are achieved; miR-31-5p normally inhibits myocardin [190].

Secretion of metalloproteinases and ECM components by VSMCs can also become perturbated, in turn disturbing physiological ECM turnover. The process involves the increased production of constitutively expressed MMP (for example MMP2), as well as the upregulation of MMPs associated with inflammation (MMP9) [191]. VSMCs can also secrete inflammatory cytokines themselves (TNF, IL-1β, IL-6) along with additional chemotactic factors that attract inflammatory cells to the area [192,193]. This contributes to VSMC pathology by triggering necroptosis, a form of apoptosis associated with inflammation. Certain VSMC phenotypes are also enriched in ECM proteins, reflecting an increased VSMC-mediated collagen secretion, which although triggered in an effort to replace wall structure, cannot adequately reinstate aortic wall contractility [194,195]. Release of ROS, due to the stimulation and upregulation of ROS-producing enzymes, such as nicotinamide adenine dinucleotide phosphate oxidase 4 (NOX4) and inducible Nitric Oxide synthase (iNOS) [196], is an additional hallmark of aneurysm development and contributes to a variety of pathological VSMC states, including inflammation and apoptosis [197]. Inhibition of these enzymes ameliorates aneurysm development in experimental models [198].

Finally, VSMC senescence is another key aspect of AA pathophysiology, evident with increasing age. Senescent VSMCs are characterized by DNA damage along with telomere shortening, epigenetic changes and abnormal protein expression, trafficking and degradation (proteostasis). Senescent VSMCs can be identified by markers such as Senescence-associated beta galactosidase (SA-β-gal), p21 and p16 along with additional factors that generally comprise the senescent-associated secretory phenotype (SASP) [199,200]. SASP is further characterized by a decrease in deacetylase antioxidant enzymes, including sirtuins (Sirtuin1) [193], with autophagic mechanisms, normally triggered by increased oxidative stress, impaired as well. These events further augment oxidative stress [201] and accelerate VSMC senescence, cellular death and eventually aneurysm progression [202]. Moreover, though VSMC apoptosis can serve a physiological role, it is exaggerated under pathologic conditions, contributing to disease [193] (Table 5).

### 4.4. Inflammation

A prominent aspect of aneurysm development and common driving factor for many aspects of its pathophysiology is vascular wall inflammation, most extensively observed in AAA. It can cause many deleterious events, including VSCM phenotype switch and death as well as aortic wall remodeling owing to the secretion of MMP and other protease enzymes by inflammatory cells. A variable inflammatory cellular infiltrate can be usually observed in AA specimens, accumulating as a result of various processes, including ECM degradation, intraluminal thrombus (ILT) formation and VSMC dysfunction, eventually generating a localized, inflammatory microenvironment. Normally, an inflammatory cascade is usually initiated after recognition of an antigenic substance by resident macrophages, dendritic cells and other antigen-presenting cells (APC); though resident macrophages are often found in the aortic wall under physiological conditions, most pathologic macrophages originate from circulating monocytes [203]. TLR4 upregulation in local endothelial cells and VSMCs then allows for recognition of local particles capable of initiating an inflammatory response [204].

Various macrophage populations have been identified within growing aneurysmal tissues, including M1 macrophages mainly found in the adventitia and propagating inflammation as well as M2 macrophages involved in cellular recruitment, angiogenesis, and ECM deposition (anti-inflammatory) [205]. CD45+ F4/80+ vascular macrophages can also contribute to vascular wall inflammation via surface OR activation by components of atherosclerotic plaques (octanal) and endogenous or exogenous TLR ligands [54]. Neutrophils contribute to vascular wall inflammation mainly via phagocytosis, degranulation and formation of neutrophil extracellular traps (NET). NETs are net-resembling structures protruding from neutrophil cell membranes, harboring proteases that can further damage the aortic wall and induce inflammatory cell recruitment [206]. Natural killer (NK) cells can also aggravate injury via cytotoxicity pathways [207] targeting cells such as VSMCs as well as aggravating atherosclerotic changes [205,208]. On the other hand, invariant natural killer T (iNKT) cells stimulated by the glycolipid antigen α-Galactosylceramide, have been shown to mitigate experimental, angiotensin-mediated aneurysm expansion via reduction of the inflammatory cell infiltrate and the induction of M2 macrophage phenotypes [209].

Cells of the adaptive immunity have also been identified in AA [182]. CD4+ Th1 and Th17 cells upregulate macrophage activity via secretion of TNF and Interleukin-2 (IL-2) or Interleukin-17 (IL-17), respectively, as well as modulate collagen synthesis and secretion via TNF [210]. On the other hand, Th2 groups can induce VSMC apoptosis via FS-7-associated surface antigen (Fas)–Fas ligand (FasL) interactions [205,211]. Finally, Treg groups can mitigate vascular wall inflammation, as evident by secretion of TGFβ, Interleukin-10 (IL-10), Interleukin-35 (IL-35) [205], and Forkhead box protein 3 (FOXP3). FOXP3 acetylation, in particular, is closely associated with Treg function. Reduced FOXP3 acetylation, carried out by the deacetylase SIRT1, facilitates FOXP3 proteolytic degradation and is associated with functional deficiency in Treg cells. While this could propagate inflammatory wall damage owing to decreased Treg functionality [212], SIRT can also promote Treg survival via stabilization of the membrane Notch receptor under conditions of caloric restriction [213].

Assembly of NLRP3 and Absent in melanoma 2 (AIM2) inflammasomes [214] also contributes to injury, induced by cellular debris. This is observed after upregulation of individual inflammasome components in aneurysmal tissue [215], including the apoptosis-associated speck-like protein with a caspase recruitment domain (ASC) component, caspase-1 and IL-1β [216]. Inflammasome assembly can also be triggered by surface ORs in vascular macrophages [54]. In general, inflammasomes can play a role during the early phases of aortic wall inflammation, induce MMP9 activation, aggravating inflammation, ECM degradation and thus, vascular wall injury [53,56,205].

This ongoing inflammation can also induce processes related to angiogenesis via upregulation of hypoxia inducible factor-1 alpha (HIF-1α) [217]. Downstream effectors of HIF–1α, including vascular endothelial growth factor (VEGF), stromal-derived factor 1 (SDF1), angiopoietin 1 (Ang1), angiopoietin 2 (Ang2), and platelet-derived growth factor B (PDGFB) [218] can then induce angiogenesis [219]. Additional cytokines can induce angiogenesis, including mast cell-derived protease-4 (mMCP-4) and various type I interferons (IFNI) [220]. Microvessels formed in this manner further allow monocyte/macrophage infiltration within the wall, augmenting inflammation [221] (Table 6).

### 4.5. Platelets and Intraluminal Thrombus

Sites of damage within the aortic wall, due to the ongoing ECM degradation along with disturbance of normal laminar flow [223], create spatial conditions that favor formation and attachment of an intraluminal thrombus (ILT) at sites of active inflammation and angiogenesis. Thrombus attachment prevents oxygen and nutrient diffusion within the wall, thus inducing and propagating local angiogenesis, inflammatory cell infiltration and MMP activation, processes that each exacerbate one another [6]. ILT usually comprises a fibrin-based structure with platelets, red blood cells (RBC), leukocytes and additional molecules. These include von Willebrand factor (vWF) produced by platelets (α-granules) and endothelial cells (Weibel–Palade bodies, WPB) [224], tissue plasminogen activator (tPA) and plasminogen activator inhibitor (PAI) [225]. ILTs are composed of a luminal layer exposed to the circulation, aiding in the activation and aggregation of circulating platelets via αΙIbβ3 integrin–fibrinogen interactions [226] and an abluminal layer in contact with the vascular wall [227]. Mature thrombi also possess canaliculi, enabling cells and other circulating factors to enter the structure [228]. They can have both negative and positive effects on aneurysm progression, though negative aspects usually predominate, rendering antiplatelet medication useful for AA management [229].

Endothelial dysfunction creates a stimulus for the attachment and development of ILTs, facilitating the initial attachment of circulating platelets; disequilibrium between pro-coagulation and anti-coagulation factors is an inciting factor for this aggregation [229], while endothelial damage as an inciting factor has been reported as well [230]. Furthermore, platelets are also more reactive, mainly due to the disturbance in flow and hemodynamic conditions [231]. In addition, aggregation can occur even without prior platelet activation, due to the local hemodynamic conditions including reduced flow velocity and vortex flow. Platelets aggregating in the ILT will then contribute to formation, expansion and proteolytic characteristics of the generated thrombus [232]. As opposed to other thrombus types, ILTs in AA are not characterized by vascular mesenchymal stem cell (vMSC) colonization or surface reendothelialization via adhesion of other circulating progenitors [233]. This is due to the local release of proteases, including neutrophil-derived elastase and MMP9 [234].

Platelet aggregation, along with P-selectin and P-selectin glycoprotein ligand-1 (PSGL-1) interactions on platelet and neutrophil surfaces, respectively, along with interactions between additional receptors, allow for the adhesion and activation of neutrophils with subsequent generation of NETs [235]. Neutrophils undergo cell death upon contact with fibrin, triggering the release of MMPs, inflammatory cytokines and other enzymes [236]. Macrophages within the ILT canaliculi can also secrete inflammatory cytokines and MMPs, while macrophages on the luminal ILT surface, possessing a predominantly anti-inflammatory phenotype, can contribute to the mitigation of the observed inflammation via secretion of associated anti-inflammatory cytokines [237,238,239].

The effects of platelets upon the structure of the aneurysmal sac and progression of disease can also differ depending on the quality of ECM collagen; relevant studies have shown a propensity for circulating platelets to adhere to areas with normal collagen structure in the aneurysm, which are interspersed among areas of abnormal collagen [230]. Platelets can also secrete factors that aid in the recruitment of inflammatory cells (monocyte chemoattractant protein-1-MCP-1, β2-microglobulin) [240,241], VSMC apoptosis (Platelet-derived growth factor-PDGF) [180] and aortic wall hypoxia [242] as well as proteases that contribute to ECM degradation (MMP9) [243]. Conversely, some studies have shown certain platelet-derived factors to be associated with protective effects, as is the case with Platelet factor-4 (PF4) ameliorating TAA progression in murine models via stabilization of the endothelial cell lining [244].

ILTs in aortic aneurysm are thus biologically active, facilitating the secretion of various inflammatory cytokines (interferon gamma (IFNγ), IL-1α), platelet-derived factors (PF4), Platelet-derived growth factor (PDGF)), TGFβ, MMPs (MMP8, MMP9) as well as various neutrophil products such as urokinase plasminogen activator (uPA), proteinase-3, cathepsins, myeloperoxidase (MPO) and elastase, within both the aortic wall and lumen. Contrary to other types of thrombi, ILTs do not resolve temporally due to the continuous presence of neutrophils inducing endothelial injury/detachment from the subendothelial matrix, via neutrophil elastase inducing fibronectin degradation, along with other proteases [245]. Local red blood cell (RBC) lysis in the ILT leads to release of heme and iron, further contributing to thrombus propagation, most likely due to exacerbation of oxidative stress [246]. Eventually, ECM degradation and VSMC dysfunction/apoptosis are both aggravated, contributing to propagation of the aneurysm itself [247].

Once the ILT reaches critical mass, it can reduce or prevent oxygen and nutrient diffusion, creating hypoxic conditions that stimulate production of HIF-1α [223], though in general, the infrarenal aorta seems to be more susceptible to intramural hypoxia [248]. Apart from hypoxia, HIF-1α can be upregulated in response to other stimuli as well, including ROS and growth hormones [217]. HIF-1α signaling pathways have been identified in macrophages, VSMCs [220] and adventitial aortic fibroblasts, implicating all these cell types in the associated angiogenic and remodeling processes [249,250], leading to the activation of downstream effectors (VEGF, SDF1, Ang1, Ang2, PDGFB) that eventually induce angiogenesis [251]. Polymorphisms in both HIF-1α and VEGF genes are responsible for increasing predisposition to both aneurysm disease and atherosclerosis [252], while higher VEGF levels correlate with aneurysm occurrence and size as well as risk for acute events such as rupture. Pathological intramural angiogenesis is associated with both pro-angiogenic factors (Ang1, Ang2) and anti-angiogenic factors (TSP1) [253,254]. As a result, though newer microvessels are generated, these vascular tubes are often structurally immature with increased permeability [219,255,256,257] (Table 7).

#### Olfactory Receptors, Platelets and Intraluminal Thrombi

Platelets and their behavior during AA can be further modified through activation of ORs present on their surface, as has been previously detailed. OR2L13, in particular, is upregulated in response to biomechanical stressors such as turbulent flow. Platelets in patients with aortic aneurysm are already more reactive and prone to secretion of MMP, as already iterated; under turbulent conditions, platelet budding from megakaryocytes is upregulated as well [258]. Once these new platelets are subjected to turbulence, they become biomechanically active themselves, exhibiting one additional characteristic: translocation and increased distribution of surface OR2L13 receptors, although *OR2L13* gene transcription itself does not change [59]. OR2L13 has been experimentally stimulated with various compounds, including vanillin, myrrh, frankincense and carvone, with (−) carvone enantiomers having a more potent effect [59]. Carvone is a volatile, natural monoterpene with low molecular weight and water solubility, while recent studies also point to anti-inflammatory functions. When chemically modified to reduce volatility, carvone derivatives exhibit various anti-inflammatory functions, including inhibition of LPS-induced iNOS activation as well as modulation of proteolysis and subsequent activation of pro-IL-1β [259].

**Figure 3:**
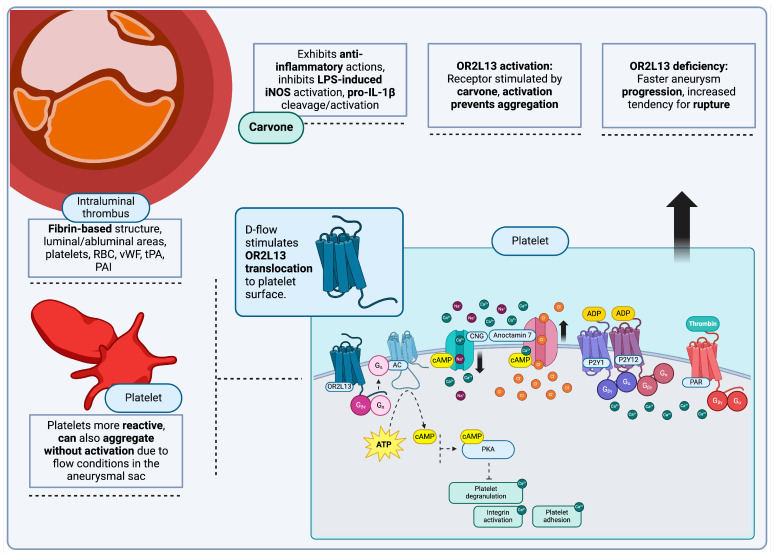
Signaling mechanisms associated with OR2L13 activation in the general scope of aortic aneurysm. Platelets during disturbed hemodynamic flow can aggregate, or they can be more easily activated. Disturbed flow leads to translocation of OR2L13 receptors on the platelet surface. Experimentally, these receptors can be activated by substances such as carvone, which will then inhibit platelet degranulation and integrin activation, allowing for platelet aggregation to be prevented. Carvone can be subjected to chemical modifications that reduce volatility, which further gives such compounds anti-inflammatory capabilities such as ability for the inhibition of LPS-induced iNOS and the inhibition of pro-IL-1β cleavage/activation. Carvone-mediated OR2L13 activation antagonizes the platelet adhesion, integrin activation and platelet degranulation associated with stimulation of P2Y1/P2Y12 and PAR receptors by ADP and thrombin, respectively. In addition, experimental deficiency of OR2L13 leads to faster rates of aneurysm progression along with a higher tendency for growth and earlier aneurysm rupture (created in BioRender.com, Accessed on 18 November 2024) [59,232,259]. AC, Adenylate cyclase; ADP, Adenosine diphosphate; cAMP, cyclic Adenosine monophosphate; PKA, Protein kinase A; ATP, Adenosine triphosphate; CNG, Cyclically activated nucleotide gated channels; Na^+^, Sodium; Ca^2+^, Calcium; Cl, Chloride; ROS, Reactive oxygen species; OR2L13, Olfactory receptor family 2 subfamily L gene 13; cAMP, cyclin Adenosine monophosphate; Cl, Chloride; Na, Sodium; PKA, Protein kinase A; PAR1, Protease-activated receptor type 1; RBC, Red blood cell; vWF, von Willebrand factor; tPA, tissue Plasminogen activator; PAI, Plasminogen activator inhibitor; D-flow, Disturbed flow; LPS, Lipopolysaccharide; iNOS, inducible Nitric oxide synthase; pro-IL-1β, pro-Interleukin one-beta. Figure 3. Created in BioRender. Stougiannou, T. (2025) https://BioRender.com/c32e126 (accessed on 1 November 2024).

Upon OR activation, increased cAMP concentrations are produced via adenylate cyclase (AC) stimulation, leading to CNG channel activation. This, in turn, triggers intracellular Ca^2+^ transients and as a result, Ca^2+^-induced activation of anoctamin 7, a chloride (Cl^−^) channel. Cl^−^ efflux will then inhibit platelet aggregation, Ca^2+^-mediated integrin activation and platelet degranulation, via the cAMP-mediated activation of PKA [59,260]. This is further corroborated by an increased tendency for aneurysm progression with earlier rupture, observed in OR2L13-deficient mice [59] (Figure 3).

### 4.6. Endothelium

Endothelial cells have multiple effects on the pathobiology of aortic aneurysm. As with VSMCs, various endothelial phenotypic groups have been identified based on their localization with respect to the aortic wall (some endothelial cells are found within the adventitia) and the amounts of cell–cell and cell–ECM junctions. The latter reflects their propensity for mobilization or migration [182]. Phenotypic switch of endothelial cells can also occur (EndMT); it is mainly driven by TGFβ signaling, oxidative stress and pro-inflammatory cytokines such as IL-1β [261]. During this time, cells lose their epithelial characteristics and acquire phenotypes observed in cells of mesenchymal origin, including contractility, expression of VSMC-specific markers and collagen genes as well as an increased tendency for migration, evident by loss of endothelial cell–cell junctions. Eventually, this facilitates entry of circulating immune cells and factors in the aortic wall [262].

Disruption of aortic endothelial barrier function is one of the earliest events during AA occurrence. The barrier is normally modulated by angiogenic factors such as vascular endothelial growth factor-A (VEGF-A), Ang1, Ang2 and inflammatory cytokines (histamine, bradykinin) [263]. Barrier function disruption can occur due to a dysregulation in the proteins comprising focal adhesions (cell adhesions between endothelial cells, ECM and the cytoskeleton) and cell-cell adhesions such as vinculin, vascular endothelial (VE)-cadherin, p120 catenin and claudin 5 [264]. The endothelial barrier can also be affected by proteolytic enzymes such as A disintegrin and metalloproteinase 17 (ADAM17). These enzymes, apart from ECM degradation and inflammation, regulate cell–cell adhesion via proteolytic shedding of transmembrane proteins such as VE-cadherin (adherens junctions), junctional adhesion molecule A (tight junctions) and claudin-5 (tight junctions). ADAM17 is secreted by both endothelial cells [265] and VSMCs [266], promoting VE-cadherin cleavage and cell–cell junction disruption in the endothelium as well as phenotypic switch and apoptosis in VSMCs [265].

Roundabout 4 (ROBO4) is transmembrane protein receptor usually expressed on the surface of endothelial cells, important for maintenance of the vascular endothelial barrier. ROBO4 activation prevents an increase in VEGF-induced vascular permeability, either via VEGFR-2 internalization or interaction with the transmembrane protein Unc5B, eventually causing VEGF/VEGFR2 signaling downregulation [267]. ROBO4 also downregulates TNF-induced vascular permeability via association with TNF-associated receptor factor 7 (TRAF7) (ROBO4–TRAF7). ROBO4–TRAF7 complexes will then prevent VE-cadherin redistribution on the endothelial cell surface, a phenomenon normally induced by TNF–TNFR interactions [268]. ROBO4 can also suppress endothelial phenotype switch (EndMT) [264,269,270,271]. Various *ROBO4* variants or mutations have been associated with TAA, resulting in endothelial barrier dysregulation with all of its associated sequelae [272].

Flow and shear stress can also have varying effects on vascular endothelial cells, affecting aspects of endothelial geometric alignment and influencing gene expression. Normal shear stress associated with pulsatile flow modulates membrane potential [273], facilitates production of vasodilatory factors (prostacyclin, nitric oxide (NO)) and tPA, which prevent thrombogenesis and inflammation. On the other hand, disturbed flow facilitates the secretion of pro-inflammatory cytokines such as MCP-1, PDGF and endothelin-1, promoting leukocyte infiltration within the aortic wall [274].

Endothelial cells possess various types of mechanosensory molecules, including the VE-cadherin-Platelet endothelial cell adhesion molecule 1 (PECAM1)–VEGFR2/VEGFR3 junctional protein complex. PECAM1 stimulation triggers VEGFR2/VEGFR3 activation, allowing for shear stress mechanosensation in lymphatic and vascular endothelial cells [275] and stimulating shear stress-induced vascular remodeling [276]. Recent studies have pointed to a relevant role for the shear stress sensor junctional protein, angiomotin-like protein 2 (AmotL2), normally stimulated by laminar flow and interacting with VE-cadherin and actin filaments (cytoskeleton) [277]. AmotL2 facilitates appropriate endothelial cell alignment and morphology. Under conditions of reduced mechanosensory stimulation, there is reduced actin polymerization, allowing for angiomotin to bind Yes-associated protein homolog 1 (YAP)/Transcriptional coactivator with PDZ-binding motif (TAZ), inhibit YAP/TAZ–Transcriptional enhanced associate domain (TEAD) complex formation and as a result, gene expression [278]. Absence of AmotL2 has been associated with inflammation in the tunica intima and aortic aneurysm in experimental studies, possibly due to the resulting loss of endothelial barrier integrity and upregulation of inflammatory cytokines in affected endothelial cells (IL-6) [277]. The mechanosensitive ion channel Piezo1 also associates with VE-cadherin and PECAM1, maintaining Ca^2+^ flow and normal actin filament configuration in response to flow variations [279]. Under conditions of disturbed flow, Piezo1 stimulates activation of the junctional VE-cadherin-PECAM1–-VEGFR2/VEGFR3 protein complex, leading to the upregulation of NF-κB in affected cells and as a result, inflammation, which can also contribute to atherosclerosis [280].

Finally, oxidative stress can contribute to aortic aneurysm pathogenesis as well; uncoupling of endothelial nitric oxide synthase (eNOS) from tetrahydrobiopterin (BH_4_) leads to production of superoxide instead of nitric oxide (NO). BH_4_ availability is associated with absence of dihydrofolate reductase (DHFR) owing to a lack of BH_4_ regeneration [281]. However, NO itself also contributes to AA progression, via induction of proteins such as extracellular matrix metalloproteinase (MMP) inducer (EMMPRIN) [282], leading to the activation of MMP13 [283]. EMMPRIN has been identified in higher amounts in the aortic wall of Marfan specimens, implicating the protein in the aortic wall injury associated with TAA in such cases [282]. There is also a reported increase in associated markers of oxidative stress in AAA patients, including a systemic rise in malondialdehyde (MDA) as well as a local increase in superoxide generated by NADPH oxidase (NOX) [284,285] (Table 8 and Table 9).

## 5. Discussion

Aortic aneurysm is a multifaceted pathobiological process, with varying etiological factors, many of which are of genetic etiology; genetic variations and mutations are associated with both TAA and AAA, though in the case of TAA, more syndromic cases are reported or identified. In the case of AAA, on the other hand, and though many of the genes affected in TAA also seem to be affected in AAA, there is a predilection for gene variants associated with inflammatory pathways [5]. Furthermore atherosclerosis, though initially considered a different disease entity merely sharing features with aortic aneurysm, has been shown to contribute to both TAA and AAA [121]. Though atherosclerosis and aortic aneurysm are thus different disease entities, they exhibit similarities and common disease mechanisms, particularly with regard to the vascular wall inflammation and dysregulated lipid trafficking contributing to disease progression [286], both shown in recent research to possess a causal relationship with AAA development [159]. On the contrary, diabetes mellitus (DM), a risk factor for atherosclerosis, has been negatively associated with AA development [121,284,287].

Sirtuins possess a mixed functionality with regard to aneurysmal pathobiology; though they prevent ROS-mediated damage to the aortic wall and enhance survival of Treg cells, they contribute to the deacetylation of FOXP3. ROS normally exacerbate inflammatory injury in the tunica media and affect endothelial cell function in the intima. Though acetylation and deacetylation of FOXP3 is part of the physiological control of intracellular FOXP3 protein levels [288], abnormally high rates of FOXP3 deacetylation can interfere with Treg functionality and thus their ability to suppress excessive inflammatory responses. Reduced numbers of FOXP3+ Treg cells with associated higher SIRT1 expression levels have in turn been correlated with AAA development in relevant studies, while treatment with a SIRT1 inhibitor restores Treg function [212]. However, this effect seems to be specific to SIRT1 [288]. This indicates not only the complex contribution of sirtuins to aneurysm pathobiology as a whole but may also complicate pharmacological treatment, particularly with regard to the use of pharmacological SIRT1 agonists [289] versus use of SIRT1 antagonists [212] for AA treatment. Furthermore, the specificity of SIRT1 for FOXP3 acetylation may allow for the concurrent use of sirtuin agonist and inhibitors to target pathobiological pathways [212].

The main treatment for AA is surgical repair, indications for which can differ depending on classification. In TAA, surgical repair is usually warranted once diameter reaches 5.5 cm, although in case of genetic disease, the limit can be lower [2]. For AAA, on the other hand, repair can be attempted at a threshold equal to or greater than 5.5 cm for men and 5.0 cm for women [2]. Finally, in aneurysms spanning both the thoracic and abdominal aorta (TAAA), indications are not as clear, mostly due to lack of appropriate and clear evidence; however, presence/risk for acute dissection, a diameter greater than 6.0 cm (or 5.5 cm if carried out in specialized centers), rapid growth, penetrating atherosclerotic ulcer and aortic valve insufficiency are some of the associated indications [290,291,292]. Open surgical repair has been a common method for the repair of these aneurysms, although for some entities such as TAAA, it can often be associated with catastrophic complications such as massive hemorrhage, cardiac arrest, multisystem organ failure or conditions that increase morbidity (paraplegia, renal failure) [293]. However, improvements in surgical science and the advent of new operative techniques have aided in the reduction of catastrophic complications and improved survival after the operation [2,294]. Endovascular repair is another option for surgical repair, with various types of endovascular devices (multibranched, fenestrated, multibranched and fenestrated) [295] as well as custom-made endovascular devices used [296], though success can be affected by local anatomy [297,298]. Endovascular repair is commonly utilized for aneurysms in the abdominal aorta [299], though it can also be used to tackle aneurysms in the aortic arch [300] and ascending aorta [301]. In addition, the associated lack of need for aortic cross-clamping often leads to better outcomes with regard to post-operative neurological sequelae, morbidity and mortality [296].

Various ectopic olfactory receptors have been found in different locations other than the olfactory mucosa, including receptors relevant to the function of the cardiovascular system. Two main receptors have been recently investigated with regard to AA pathophysiology; while OR6A2 (Olfr2) is associated with vascular wall inflammation, atherosclerotic plaque size and AA progression, OR2L13 (Olfr168) is associated with prevention of platelet aggregation, and in experimental studies, deficiency in this receptor is associated with faster aneurysm progression and increased risk of rupture. OR-mediated aortic wall inflammation thus exacerbates not only atherosclerosis, owing to oxLDL-mediated effects, but also represents an additional cause of vascular wall inflammation and contributes to aortic aneurysm formation [53,64].

While OR6A2 (Olfr2) is endogenously activated by atherosclerotic and inflammatory ligands, studies so far have only shown only an upregulation in the OR2L13 (Olfr168) receptor in response to disturbed hemodynamic conditions, as the latter has been usually activated experimentally with substances like carvone [53,59,173]. Carvone, when chemically modified, can have anti-inflammatory actions; these effects, along with possible actions on platelet aggregation, may render such substances useful for the pharmacological treatment of AA, in addition to the pharmaceutical formulations already in use. Inhibition of the OR6A2 (Olfr2) receptor may also have a role against aortic atherosclerosis, vascular wall inflammation and the ECM/VSMC disruption frequently observed in these cases, as relevant studies with OR6A2 (Olfr2)-deficiency show a halt in the progression of aortic dilatation [65,173]. While the function of the OR2L13 (Olfr168) receptor with regard to platelet aggregation and its association with aneurysm has been examined, it may be useful to further investigate its function in relation to parameters such as aortic wall inflammation and inflammatory cell recruitment, as platelet activation can also lead to the secretion of factors that contribute to inflammation (MCP-1, β2-microglobulin) [240,241]. In addition, evaluating the function of other, similar receptors found on the platelet surface (OR2W3, OR2B6) [59] in the context of aortic aneurysm and vascular wall inflammation may provide additional context regarding platelet activation in these cases.

Among other receptors identified with functions relevant to the cardiovascular system, OR10J5 (Olfr16) has been found in human aortic samples [35]; it would thus be useful to further evaluate its role with regard to aneurysm development. Both OR10J5 and OR6A2 (Olfr2) have been associated with endothelial cells [35,53]. As pathobiological disruptions in normal endothelial function, including endothelial barrier function, are often some of the first events inciting development of AA pathology [263], it would be useful to evaluate the effects of both of these receptors in association with endothelial pathology contributing to aneurysm development. Evaluation of these receptors and their function may thus aid in the identification of new pathological pathways or increase knowledge regarding current pathways, allowing for development of new treatment methods combined with or used during surgical repair of AA.

## 6. Conclusions

The initiation and propagation of aortic aneurysm, though complex, can be grouped according to disruption in key processes/areas, including dysregulation in ECM homeostasis, lipid metabolism and trafficking within the circulation as well as atherosclerosis, disruption in the survival and many functions of VSMCs, platelet aggregation and thrombus (ILT), angiogenesis and localized inflammation. Many of these processes exist simultaneously, each individually contributing to propagation of aortic wall injury and all together further inducing and aggravating wall inflammation and thus, injury. Among the various causative factors and mechanisms, a role for ectopic ORs in AA has been uncovered, with experimental studies associating specific receptors with disease progression. In line with these recent discoveries, additional research into other ectopic ORs may further enrich knowledge of AA pathophysiology as well as create or contribute to new treatment pathways.

## Figures and Tables

**Table 1:** Odorant olfactory receptors (ORs) identified in cells and tissues relevant to the cardiovascular system. OR proteins encoded by the corresponding *OR* genes found in humans are written with the naming convention described early in Section 2, while the protein product of the corresponding gene ortholog found in mice is mentioned in parentheses for each. OR51E1, Olfactory Receptor family 51 subfamily E gene 1; MCFA, Medium chain fatty acid; OR51E2, Olfactory Receptor family 51 subfamily E gene 2; VSMC, Vascular smooth muscle cells; SCFA, Short chain fatty acids; JGA, Juxtaglomerular apparatus; Olfr78, Olfactory receptor 78; OR10J5, Olfactory Receptor family 10 subfamily J gene 5; HUVEC, Human umbilical vein endothelial cells; AKT, PKB or Protein Kinase B; Gpr41; G-protein coupled receptor 41; Gpr43, G-protein coupled receptor 43; Olfr2, Olfactory receptor 2; OR6A2, Olfactory receptor family 6 subfamily A gene 2; CD45, Cluster of differentiation 45; CD31, Cluster of differentiation 31; oxLDL, oxidized Low-density lipoprotein; ROS, Reactive oxygen species; LPS, Lipopolysaccharide; NLRP3 inflammasome, NLR family pyrin domain containing 3 inflammasome; IL-1β, Interleukin one beta; IL-1α, Interleukin one alpha; IL-6, Interleukin-6; TNF, Tumor necrosis factor; OR2L13, Olfactory receptor family 2 subfamily L gene 13; D-flow, Disturbed flow.

Receptor	Characteristics/Function	References
OR51E1 (Olfr558)	Fetal, adult cardiomyocytes; negative inotropic, chronotropic effects	[37]
	**Agonists:** MCFA (nonanoic, dodecanoic, tetradecanoic), some endogenously stored and secreted by epicardial fat tissue	
OR51E2 (Olfr78)	VSMCs in resistance vessels of heart, skeletal muscle, skin; modulation of arterial pressure, tissue perfusion	[47,49,50,51,52,63]
	VSMCs in the JGA afferent arteriole, counterregulation of hypotension mediated via propionate-induced activation of Gpr41, Gpr43 (in murine models used to evaluate function of the Olfr78 receptor)	
	**Agonists:** SCFA (acetate, propionate), androgens, β-ionone	
OR10J5 (Olfr16)	Human aorta, coronary arteries, HUVEC; microtubule disassembly/cortical rim actin assembly, cell–cell junction disruption, endothelial cell migration (AKT signaling), angiogenesis	[35,46]
	**Agonists:** Lyral, α-cedrene	
OR6A2 (Olfr2)	CD45+ F4/80+ vascular macrophages (aorta), CD45- CD31+ endothelial cells (aorta), CD45- CD31- VSMCs (aorta)	[53,54,64,65]
	ROS production in cytosol, mitochondria, NLRP3 inflammasome activation, release of IL-1β, IL-1α, IL-6 and TNF, deficiency mitigates aortic aneurysm formation, remodeling and inflammation (murine models)	
	**Agonists:** Octanal	
OR2L13 (Olfr168)	Platelets, megakaryocytes; stored in α-granules, co-localizes with P-selectin, upregulated by D-flow	[59]
	Counterregulates platelet aggregation induced by other mechanisms, deficiency leads to increased aneurysm growth, early rupture (murine models)	
	**Agonists:** vanillin, myrrh, frankincense, carvone ((−) enantiomer producing stronger effect), octanoate	

**Table 2:** Main genes/gene groups associated with thoracic (TAA) and abdominal aortic aneurysm (AAA), along with the pathobiological processes they can contribute to, in case of mutations or genetic variations. ECM, Extracellular matrix; VSMC, Vascular smooth muscle cells; TGFβ, Transforming growth factor beta; SAM, S-adenosylmethionine; DNA, Deoxyribonucleic acid; RNA, Ribonucleic acid; Acta1, smooth muscle actin α2; Myh11, smooth muscle myosin heavy chain 11; Mylk, myosin light chain kinase; Flna, Filamin A; Tgfbr1, TGFβ receptor type I; Tgfbr2, TGFβ receptor type II; Tgfb2, Transforming growth factor β2; Smad3, Mothers against decapentaplegic drosophila homolog 3; Smad4, Mothers against decapentaplegic drosophila homolog 4; Lox, Lysyl oxidase; Bgn, Biglycan; Eln, Elastin; Col3a1, Procollagen type III α1; Mat2a, Methionine adenosyltransferase II α; SAM, S-adenosylmethionine; TAD, Thoracic aortic disease; AD, Aortic Dissection; Arih1, Ariadne drosophila homolog 1; Fbn1, Fibrillin-1; Il6r, Interleukin-6 receptor; Sort1, Sortilin-1; Lrp1, Low-density lipoprotein receptor-related protein 1; Ldlr, Low-density lipoprotein receptor; Smyd2, MYND (myeloid-nervy-DEAF1) domain containing 2; Erg, Transcriptional regulator ERG; Dab2ip, DAB 2 interacting protein; Cdnk2b, Cyclin-dependent kinase inhibitor 2b; Cdkn2b-As1, Cyclin-dependent kinase inhibitor 2b-Antisense ribonucleic acid (RNA); Anril, Antisense non-coding ribonucleic acid (RNA) in the INK4 locus; Mmp, Matrix metalloproteinase; Il6, Interleukin-6; Il1α, Interleukin-1 alpha; Il1β, Interleukin-1 beta.

Gene	Functional Group	TAA (AD)/AAA	References
*Fbn1*, *Lox*, *Bgn*, *Eln*, *Col3a1*, *Lrp1*	ECM homeostasis	TAA	[5,83,84,85,105]
*Col3a1*, *Fbn1*, *Lrp1*, *Erg*, *Mmp*		AAA	[5,93,95,105,108]
*Acta1*, *Myh11*, *Mylk*, *Flna*, *Lrp1*	VSMC	TAA	[5,103]
*Lrp1*, *Smyd2*		AAA	[5,103,106]
*Tgfbr1*, *Tgfbr2*, *Tgfb2*, *Smad3*, *Smad4*	TGFβ signaling	TAA	[5]
*Tgfbr1*, *Tgfbr2*		AAA	[96]
*Mat2a*	SAM metabolism	TAD (AD), TAA	[5,86,87]
*Arih1*	Cytoskeleton	TAD (AD), TAA	[5]
*Sort1*, *Ldlr*	Lipid trafficking/metabolism	TAA, AAA	[5,112,113]
*Cdkn2b-As1/Anril*, *Cdkn2b*, *Dab2ip*, *Il6r*, *Smyd2*, *Erg*, *Il6*, *Il1α*, *Il1β*	Inflammation	AAA	[5,96,100,101,107,108]
*Erg*	Endothelial dysfunction	TAA, AAA	[5,108,109]

**Table 3:** Comparison of non-genetic etiological factors associated with thoracic (TAA) and abdominal aortic aneurysm (AAA) disease. HTN, Hypertension; CoA, Coarctation of the Aorta; BAV, Bicuspid aortic valve; cAAA, Congenital abdominal aortic aneurysm; GCA, Giant cell arteritis; TA, Takayasu arteritis; SLE, Systemic lupus erythematosus; GPA, Granulomatosis with polyangiitis; RA, Rheumatoid arthritis; AS, Ankylosing spondylitis; IgG4, Immunoglobulin G4; IAAA, Inflammatory Abdominal Aortic Aneurysm.

TAA	References	AAA	References
HTN	[114]	HTN	[139]
Atherosclerosis	[121]	Atherosclerosis	[121]
Age	[120,121]	Age	[119]
Smoking	[115]	Smoking	[135]
Congenital disease (CoA, BAV)	[117,118]	Congenital disease (cAAA)	[143]
Approx. equal sex distribution, unless other risk factors present	[119]	Male sex	[119]
Cocaine	[116]	Fluoroquinolones	[141,142]
Infectious aortitis	[122]	Infectious aortitis	[122,133,134]
Non-infectious aortitis (GCA, TA, IgG4-related aortitis, SLE, sarcoidosis, GPA, RA, AS)	[81,123,124,125,127,128,129,131]	Non-infectious aortitis (TA, GCA, AS, IgG4-related aortitis)	[124,128,130,131]
		IAAA (chronic idiopathic periaortitis, idiopathic retroperitoneal fibrosis)	[132]

**Table 4:** Main processes characterizing extracellular matrix (ECM) derangements in aortic aneurysm pathophysiology. MMP, Matrix metalloproteinase; ADAM, A disintegrin and metalloproteinase enzyme; ADAM-TS, A disintegrin and metalloproteinase enzyme with thrombospondin motifs; TAA, Thoracic aortic aneurysm; AAA, Abdominal aortic aneurysm; TIMP, Tissue-inhibitor of metalloproteinase; TIMP3, Tissue-inhibitor of metalloproteinase 3; Fbn1, Fibrillin-1; TGFβ, Transforming growth factor beta; LOX, Lysyl-oxidase; VSMC, Vascular smooth muscle cell.

Component	Description	References
MMP	Various enzymes including ADAM-TS (TAA), ADAM (TAA, AAA), Cathepsin (AAA), upregulation due to inflammatory cytokines, macrophages, neutrophils	[70]
TIMP	Generally reduced expression or complete deficiency of TIMP enzymes, TIMP3 overexpression	[70,145,146]
Elastin	Elastin fragmentation, structural derangements due to genetic etiologies (*Fbn1*); TGFβ signaling disruption	[147,148]
Collagen	Disruption in normal collagen concentrations, structural derangements in triple helix structure and cross-linking (LOX)	[149,151]
Proteoglycans	Increased versican and aggrecan production (TAA), decreased versican, perlecan and aggrecan production (AAA), accumulation of fibronectin, thrombospondin, dysregulation in VSMC/ECM interactions	[152,153,154]

**Table 5:** Main pathological characteristics of vascular smooth muscle cells (VSMC), as part of the general pathobiology of aortic aneurysm. VSMC, Vascular smooth muscle cells; Klf4, Krüppel-like factor 4; Myocd, Myocardin; SRF, Serum response factor; PDGF-BB, Platelet-derived growth factor-BB; MSCs, Mesenchymal stem cells; ALDH, Aldehyde dehydrogenase; MMP2, Matrix metalloproteinase 2; MMP9, Matrix metalloproteinase 2; ECM, Extracellular matrix; TNF, Tumor necrosis factor; IL-1β, Interleukin-1 beta; IL-6, Interleukin 6; ROS, Reactive oxygen species; NOX4, Nicotinamide adenine dinucleotide phosphate oxidase 4; iNOS, inducible Nitric oxide synthase; SA-β-gal, Senescence-associated beta galactosidase; p21, Cyclin-dependent kinase inhibitor 1A (CDN1A); p16, Cyclin-dependent kinase inhibitor 2A (CDKN2A); SASP, Senescence-associated secretory phenotype.

Process	Description	References
Phenotype Switch	*Klf4* upregulation, *Myocd*, *SRF* downregulation, suppression of contractile phenotype genes, upregulation of phagocytic-like markers (PDGF-BB, oxidized phospholipids)	[179,180]
	VSMC types include contractile, inflammatory, stressed, proliferative (synthetic/contractile phenotype), fibromyocytic group, degradative (proteolytic/phagocytic gene phenotype), MSC-like, osteoblastic	[182,183,184,185,187]
	*ALDH2* deficiency prevents phenotype switch, maintains contractile characteristics	[190]
Secretory characteristics	Increased secretion of MMP (MMP9, MMP2), ECM components (i.e., collagen), inflammatory cytokines (TNF, IL-1β, IL-6)	[191,192,193,194,195]
Oxidative stress	ROS production due to upregulation of ROS-producing enzymes (NOX4, iNOS)	[196,198]
Senescence	DNA damage, telomere shortening, epigenetic changes, dysregulated proteostasis, increased production of SA-β-gal, p21, p16, decreased expression of antioxidant enzymes such as sirtuins, impaired autophagy (SASP)	[193,199,201,202]

**Table 6:** Main processes characterizing inflammatory pathways in aortic aneurysm pathophysiology, with possible connections to ectopic odorant olfactory receptor (OR) pathways. ECM, Extracellular matrix; CD45, Cluster of differentiation 45; OR, Odorant olfactory receptors; NET, Neutrophil extracellular traps; ILT, Intraluminal thrombus; NKT, Natural killer T cells; iNKT, invariant Natural killer T cells; CD4, Cluster of differentiation 4; TNF, Tumor necrosis factor; IL-2, Interleukin-2; IL-17, Interleukin-17; ECM, Extracellular matrix; Th1, T helper 1 lymphocytes; Th2, T helper 2 lymphocytes; VSMC, Vascular smooth muscle cell; FOXP3, Forkhead box protein P3; IL-10, Interkeukin-10; IL-35, Interleukin-35; Treg, T regulatory lymphocytes; Fas, FS-7-associated surface antigen; FasL, FS-7-associated surface antigen ligand; SIRT1, Sirtuin 1; NLRP3 Inflammasome, Nucleotide-binding oligomerization domain-like receptor family *(NLR)* pyrin domain containing 3 Inflammasome; AIM2 Inflammasome, Absent in melanoma 2 inflammasome; PDGFB, Platelet-derived growth factor Beta; HIF-1α, Hypoxia inducible factor 1 alpha; VEGF, Vascular endothelial growth factor; SDF1, Stromal-Derived factor 1; Ang1, Angiopoietin 1; Ang2, Angiopoietin 2; mMCP-4, mast cell-derived protease-4; IFNI, Interferon I.

Component	Description	References
Macrophage	M1 macrophages (adventitia) propagate vascular wall inflammation, M2 macrophages (anti-inflammatory) contribute angiogenesis, ECM deposition	[205]
	CD45+ F4/80+ vascular macrophages propagate wall inflammation via activation of surface ORs by atherosclerotic and pro-inflammatory ligands	[54]
Neutrophil	Phagocytosis, degranulation, formation of NETs	[206]
	NETs contribute to aortic wall damage via protease secretion and as part of the ILT	[206,222]
NKT	Stimulation of cytotoxic pathways affecting VSMC survival and aggravation of atherosclerotic changes, iNKTs antagonize inflammation via reduction of inflammatory cell infiltrate and stimulation of the M2 macrophage phenotype.	[207,209]
T cell	CD4+ Th1 (TNF, IL-2), Th17 (IL-17) stimulate macrophage activity, modulate ECM collagen concentrations, Th2 contribute to VSMC apoptosis (Fas–FasL interactions), FOXP3+ Treg (IL-10, IL-35) modulate/reduce inflammation	[182,205,210]
	FOXP3 deacetylation causes its proteolytic degradation and reduced Treg functionality, FOXP3 deacetylation carried out by SIRT1	[212]
Inflammasome	NLRP3, AIM2 inflammasomes associated with AA, formation induced by cellular debris, OR activation on surface of vascular macrophages, stimulate MMP9 activity	[56,205,215,216]
HIF-1α	Secreted in response to inflammation, implicated in signaling pathways within macrophages, adventitial fibroblasts, VSMCs; HIF-1α induces activation of downstream angiogenic effectors (VEGF, SDF1, Ang1, Ang2, PDGFB), angiogenesis also augmented by inflammatory cytokines (mMCP-4, IFNI), eventually inducing formation of microvessels in the aortic wall	[217,218,219,220,221]

**Table 7:** Main characteristics of the intraluminal thrombus (ILT) and relevant platelet functions associated with aortic aneurysm, along with possible connections to ectopic odorant olfactory receptor (OR) pathways. vWF, von Willebrand factor; VSMC, Vascular smooth muscle cell; ECM, Extracellular matrix; PSGL-1, P-selectin glycoprotein ligand-1; NET, Neutrophil extracellular trap; MMP, Matrix metalloproteinase; MMP9, Matrix metalloproteinase 9; ILT, Intraluminal thrombus; RBC, Red blood cell; Fe^2+^, Iron; tPA, tissue Plasminogen activator; PAI, Plasminogen activator inhibitor; IFNγ, Interferon gamma; IL-1α, Interleukin-1 alpha; PF4, Platelet factor 4; PDGF, Platelet-derived growth factor; TGFβ, Transforming growth factor beta; uPA, urokinase Plasminogen activator; MPO, Myeloperoxidase; MCP-1, Monocyte chemoattractant protein-1; PDGFB, Platelet-derived growth factor Beta; HIF-1α, Hypoxia inducible factor 1 alpha; VEGF, Vascular endothelial growth factor; SDF1, Stromal-Derived factor 1; Ang1, Angiopoietin 1; Ang2, Angiopoietin 2.

Component	Description	References
Platelets	Activation due to flow conditions, adhesion/attachment (activation not always required for adhesion/aggregation), aggregation via αΙIbβ3 integrin–fibrinogen interactions	[226,229,230,231]
	Secretion of factors facilitating recruitment of inflammatory cells (MCP-1, β2-microglobulin), VSMC apoptosis (PDGF), ECM degradation (MMP9) and aortic wall hypoxia, some platelet factors protective (PF4 stabilizing the endothelium in experimental TAA models)	[180,223,240,244]
Neutrophils	Adhesion due to platelet aggregation and platelet (P-Selectin)–neutrophil (PSGL-1) interactions, neutrophil activation and generation of NETs, death upon fibrin contact, release of MMP, inflammatory cytokines, neutrophil elastase (fibronectin degradation) and enzymes inducing endothelial detachment from subendothelial layers with temporal ILT propagation	[235,236]
Macrophages	Canaliculi macrophages secrete inflammatory cytokines, MMPs, and luminal anti-inflammatory macrophages that may mitigate ILT-associated inflammation	[223]
RBCs	RBC–platelet, RBC–fibrinogen binding, RBC lysis leads to heme and Fe^2+^ release, aggravation of oxidative stress, thrombus propagation	[232,246]
Soluble factors	vWF (produced by platelets, endothelial cells), tPA, PAI, IFNγ, IL-1α, platelet products (PF4, PDGF), TGFβ, neutrophil products (uPA, proteinase 3, cathepsins, MPO, neutrophil elastase)	[245]
Fibrin	Fibrinogen converted to fibrin by thrombin (coagulation cascade), fibrin monomers assemble and cross-link with other coagulation factors	[232]
HIF-1α	Secreted in response to hypoxia brought on by ILT mass, implicated in signaling pathways within macrophages, adventitial fibroblasts, VSMCs; activation of downstream angiogenic effectors (VEGF, SDF1, Ang1, Ang2, PDGFB) and inducing formation of microvessels in the aortic wall	[220,249,250,251]

**Table 8:** Main events associated with endothelium disruption, associated with aortic aneurysm pathophysiology. EndMT, Endothelial-to-Mesenchymal Transition; TGFβ, Transforming growth factor beta; VSMC, Vascular smooth muscle cells; IL-1β, Interleukin-1 beta; ROBO4, Roundabout 4; ADAM17, A disintegrin and metalloproteinase 17; VE-Cadherin, Vascular-endothelial cadherin; VSMC, Vascular smooth muscle cell; VEGF, Vascular endothelial growth factor; VEGFR2, Vascular endothelial growth factor receptor 2; TRAF7, Tumor necrosis factor (TNF)-associated receptor factor 7; TNF, Tumor necrosis factor; TNFR, Tumor necrosis factor receptor; PECAM1, Platelet endothelial cell adhesion molecule 1; VEGFR3, Vascular endothelial growth factor receptor 3; AmotL2, Angiomotin-like protein 2; Piezo1, Piezo-type mechanosensitive ion channel component 1; NF-κB, Nuclear factor kappa-light-chain-enhancer of activated B cells; eNOS, endothelial Nitric oxide synthase; BH_4,_ Tetrahydrobiopterin; DHFR, Dihydrofolate reductase; NO, Nitric oxide; EMMPRIN, Extracellular matrix metalloproteinase (MMP) inducer; MMP13, Matrix metalloproteinase 13; MFS, Marfan Syndrome; MDA, malondialdehyde.

Event	Description	References
Phenotype switch	EndMT (TGFβ, oxidative stress, IL-1β) with acquisition of mesenchymal characteristics (contractile phenotype, VSMC markers, collagen, loss of endothelial cell–cell junctions), higher propensity for migration, disruption of endothelial barrier, entry of circulating immune cells/factors (vascular wall inflammation)	[261,262]
	Endothelial phenotype switch normally suppressed by ROBO4	[269]
Endothelial barrier dysfunction	ADAM17-mediated proteolytic shedding of transmembrane proteins (VE-cadherin, junctional adhesion molecule A, claudin-5), ADAM17 secreted by endothelial cells (VE-cadherin cleavage, junction disruption) and VSMCs (phenotypic switch, apoptosis)	[264,265,266]
	ROBO4 dysregulation (gene variants, mutations) with disruption in VEGF/VEGFR2 signaling downregulation, disruption in ROBO4–TRAF7 complex formation (VE-cadherin redistribution via TNF–TNFR interactions), disinhibition of endothelial phenotype switch	[264,267,268,270,271]
Mechanosensation	VE-cadherin-PECAM1–VEGFR2/VEGFR3 stimulation by shear stress and vascular remodeling	[275]
	AmotL2 absence causes tunica intima inflammation, aortic aneurysm (endothelial barrier dysregulation) due to absence of actin filament and VE-cadherin modulation (dysregulation of endothelial shape and alignment with flow)	[277,278]
	Piezo1 (ion channel) associates with VE-cadherin/PECAM1, modulates Ca^2+^ homeostasis, cytoskeleton configuration in response to flow; under disturbed flow, the activation of VE-cadherin-PECAM1–VEGFR2/VEGFR3 leads to NF-κB upregulation and inflammation	[279,280]
Oxidative stress	eNOS uncoupling from BH_4_, leads to superoxide production (BH_4_ dependent on DHFR), NO also associated with AA progression via EMMPRIN-mediated MMP13 activation (higher in MFS aneurysmal wall)	[281,282,283,284]
	Increase in oxidative stress markers (systemic MDA, superoxide locally)	[285]

**Table 9:** Summary of odorant olfactory receptors (OR) associated with aortic aneurysm formation and results from relevant studies. Receptor names identified in humans are noted in capital letters according to the naming classification described in Section 2, while the corresponding receptor names found in murine models are noted in parentheses. cAMP, cyclic Adenosine monophosphate; PKA, Protein kinase A; Cl-, Chloride; OR2L13, Olfactory receptor family 2 subfamily L gene 13; PAR1, Protease-activated receptor type 1; TP, Thromboxane receptor; Ano7p1, Anoctamin; LPS, Lipopolysaccharide; IL-1β, Interleukin one beta; oxLDL, oxidized Low-density lipoprotein; ApoE, Apolipoprotein E; CD45, Cluster of differentiation 45; CD31, Cluster of differentiation 31; CD68, Cluster of differentiation 68; OR6A2, Olfactory receptor family 6 subfamily A gene 2; Olfr2, Olfactory receptor 2; WD, Western diet; Apoe, Apolipoprotein E; VSMC, Vascular smooth muscle cell; AAA, Abdominal aortic aneurysm; KO, Knockout; WT, Wild type; TNFα, Tumor necrosis factor alpha; IFNγ, Interferon gamma; EC, Endothelial cell; GFP, Green fluorescent protein; BMDM, Bone marrow-derived macrophage; CH, Cholesterol; HDL, High-density lipoprotein; LDL, Low-density lipoprotein; TG, Triglycerides; D-flow, Disturbed flow; MFI, Mean fluorescence intensity; MMP2, Matrix metalloproteinase 2.

Receptor	Description	References
OR6A2 (Olfr2)	Increased *Olfr2* mRNA expression in *ApoE−/−* murine aortas (relative expression of ~4) (peaking after 2 weeks of WD) compared to WT (relative expression of ~0.01), *Olfr2* mRNA expression is generally observed in *ApoE−/−* aortic vascular CD45+ F4/80 macrophages, CD45- CD31+ ECs, CD45- CD31- VSMCs with most *Olfr2* cells identified as macrophages (~ 28%), *Olfr2−/−* mice exhibit smaller atherosclerotic regions by ~50%, compared to WT	[53]
	*OR6A2* (*Olfr2* ortholog) expression correlates with macrophage content (high macrophage content associated with ~0.1 increase in mean *OR6A2* expression), atherosclerotic plaque samples generally associated with higher macrophage and *OR6A2* expression levels	[53]
	Octanal can be detected in plasma of WT mice (baseline at ~2 μM, doubles after WD) and *Apoe−/−* mice (baseline at ~7 μM, increases to ~9 μM after WD), absence of octanal in the diet indicates that it is not derived directly from diet but can be produced by lipid peroxidation of oleic acid in the atherosclerotic aorta (culturing of atherosclerotic aortic tissue with oleic acid increases octanal content to ~ 35%), endogenous human octanal concentrations exhibit positive correlation with total CH, HDL, LDL, TG levels	[53]
	In *ApoE−/−* mice, octanal treatment (4 weeks) doubles aortic atherosclerotic plaque size, induces an inflammatory response evident by a systemic increase in mean TNFα (by ~10 pg/mL), IL-1β (by ~5 pg/mL) concentrations in plasma but has no effect on total CH, HDL, LDL, TG levels; use of the odorant olfactory receptor antagonist citral induces a ~40% reduction in atherosclerotic plaque size but has no effects on systemic levels of lipids, leukocytes	[53]
	Separate treatment with LPS and octanal increases relative *Olfr2* aortic expression by ~3 (LPS) and ~1 (octanal), CD45+ F4/80 *Olfr2* vascular macrophage MFI by ~500 (LPS), *Olfr2* BMDM MFI by ~4000 (LPS) and relative *Olfr2* expression in BMDMs by ~0.5 (LPS) (octanal) compared to untreated samples; conversely, combined treatment with both LPS and octanal further increases the responses observed, with increases in relative *Olfr2* aortic expression by ~6, CD45+ F4/80 *Olfr2* vascular macrophage MFI by ~900, *Olfr2* BMDM MFI by ~5000 and relative BMDM *Olfr2* expression by ~1.5	[53]
	OR6A2 receptor expression increased in human AAA tissue (human macrophage surface) compared to controls	[64,65,173]
	Murine AAA models exhibit a peak in upregulation of the *OR6A2* ortholog, *Olfr2*, on day 7, which returns to baseline on day 28 (upregulated on the surface of pro-inflammatory monocytes, pro-inflammatory macrophages and macrophages with mixed resident/migratory behavior); no Olfr2 receptors detected on the surface of resident macrophages	[64,65,173]
	*Olfr2* KO mice exhibit reduced aortic macrophage populations, increased VSMC populations and collagen content as well as reduced pro-inflammatory cytokine levels (TNFa, IFNγ) (day 28) compared to WT; transcriptome analysis also reveals upregulation of signaling pathways related to VSMC contractile function and downregulation of signaling pathways related to leukocyte activation	[64,65,173]
OR2L13 (Olfr168)	Platelets in AAA patients are more reactive compared to those in healthy controls (increased reactivity via PAR1, TP with no significant difference in receptor density), upregulate OR2L13 (~0.7 increase in mean expression over controls) and increase its localization to the surface, especially under D-flow conditions (~20 increase in platelet surface OR2L13 MFI over controls, which increases to ~300 if platelets are subjected to D-flow)	[59]
	Various ligands have been screened for their ability to activate OR2L13 receptors (vanillin, myrrh, octanoate, frankincense and both carvone enantiomers),with (−) carvone producing the highest luciferase activity ratio (highest downstream cAMP activity) at ~3.0 compared to (+) carvone and all other ligands; activation of the OR2L13 receptors by these ligands increases intracellular cAMP, leads to Cl^−^ efflux via activation of Anoctamin channels and inhibits platelet activation and aggregation via upregulation of PKA	[59]
	(−) Carvone administration mitigates AAA growth and MMP2 activity, with both daily doses of 100 mg/kg (−) carvone and 30 mg/L aspirin producing a decrease in aortic diameter by ~ 1.6 mm (4 weeks), while the reduction in aortic MMP2 activity associated with (−) carvone administration is ~2000 compared to the ~3000 decrease observed with aspirin administration; conversely, *Olfr168* deletion augments AAA growth, evident by an increase in aortic diameter of ~0.88 mm compared to an increase of ~0.56 mm in WT mice over 4 weeks, increases mean MMP2 activity by ~5000 AU and reduces mouse survival by ~70% (4 weeks)	[59]

## Data Availability

Not applicable.
